# Development of a visualized risk prediction system for sarcopenia in older adults using machine learning: a cohort study based on CHARLS

**DOI:** 10.3389/fpubh.2025.1544894

**Published:** 2025-03-12

**Authors:** Jinsong Du, Xinru Tao, Le Zhu, Heming Wang, Wenhao Qi, Xiaoqiang Min, Shujie Wei, Xiaoyan Zhang, Qiang Liu

**Affiliations:** ^1^School of Health Management, Zaozhuang University, Zaozhuang, China; ^2^Department of Teaching and Research, Shandong Coal Health School, Zaozhuang, China; ^3^School of Nursing, Jilin University, Jilin, China; ^4^School of Public Health and Nursing, Hangzhou Normal University, Hangzhou, China; ^5^Department of Geriatics, Shandong Healthcare Group Xinwen Central Hospital, Taian, China; ^6^Image Center, Zaozhuang Municipal Hospital, Zaozhuang, China; ^7^Magnetic Resonance Imaging Department, Shandong Healthcare Group Zaozhuang Central Hospital, Zaozhuang, China; ^8^Department of Cardiovascular Medicine, Jiangxi Provincial People's Hospital, The First Affiliated Hospital of Nanchang Medical College, Nanchang, Jiangxi, China

**Keywords:** sarcopenia, risk prediction, visualized, machine learning, CHARLS

## Abstract

**Introduction:**

The older adult are at high risk of sarcopenia, making early identification and scientific intervention crucial for healthy aging.

**Methods:**

This study utilized data from the China Health and Retirement Longitudinal Study (CHARLS), including a cohort of 2,717 middle-aged and older adult participants. Ten machine learning algorithms, such as CatBoost, XGBoost, and NGBoost, were used to construct predictive models.

**Results:**

Among these algorithms, the XGBoost model performed the best, with an ROC-AUC of 0.7, and was selected as the final predictive model for sarcopenia risk. SHAP technology was used to visualize the prediction results, enhancing the interpretability of the model, and the system was built on a web platform.

**Discussion:**

The system provides the probability of sarcopenia onset within 4 years based on input variables and identifies critical influencing factors. This facilitates understanding and use by medical professionals. The system supports early identification and scientific intervention for sarcopenia in the older adult, offering significant clinical value and application potential.

## 1 Introduction

Sarcopenia has become an important health concern for the older adult, characterized by reduced muscle mass, decreased muscle strength, and gradual physical function decline (1, 2). This degenerative condition severely limits mobility, increases the risk of falls and fractures, and may lead to a decline in quality of life and higher mortality rates (3). In China, with the rapid aging process, the prevalence of sarcopenia remains high, imposing significant burdens on healthcare systems and family care (4). Identifying high-risk individuals promptly and adopting scientific interventions, such as increased protein intake and enhanced physical activity, can help maintain healthy muscle conditions and reduce the risk of sarcopenia (5, 6).

With the rapid development of big data and artificial intelligence, machine learning models based on health data have shown great potential in disease risk assessment (7, 8). These models can mine underlying patterns from large datasets, accurately identify high-risk populations, and analyze critical risk factors for diseases. For instance, Dong et al. developed a predictive model for diabetic nephropathy within 3 years among type 2 diabetes patients based on electronic health records, revealing that high homocysteine levels and poor blood glucose control were significant risk factors (9). Similarly, Wang et al. used six machine learning algorithms to predict all-cause mortality within 3 years for heart failure patients with coronary artery disease, identifying age, occupation, and nitrate use as key factors (10).

However, despite the significant achievements of machine learning in disease risk prediction, research on predicting sarcopenia in the older adult remains in its infancy. Most existing studies are based on cross-sectional data (11–13), limiting their ability to capture the dynamic process and time-dependent risk factors of sarcopenia development. Additionally, current studies primarily focus on model construction without in-depth analysis of the importance of risk indicators or features. The lack of interpretation hinders the comprehensive understanding of key influencing factors. Moreover, these studies have yet to translate their findings into practical tools, such as web-based or application-based sarcopenia assessment platforms, limiting their application in clinical practice and health management.

To address these gaps, this study utilized data from the 2011 and 2015 CHARLS databases to develop a visualized sarcopenia risk assessment system for the older adult. Various machine learning algorithms, including LightGBM, XGBoost, and AdaBoost, were employed to construct the models, with the best-performing model selected for predicting sarcopenia risk within 4 years. To enhance model interpretability, Shapley Additive Explanations (SHAP) were introduced to visualize the contribution of each feature to the prediction, increasing transparency and credibility. Finally, to further enhance the practical application value of this study, the trained model was deployed on a web platform, creating a risk prediction system for sarcopenia in older adults. This system enables medical professionals to quickly identify high-risk individuals and implement personalized prevention and intervention strategies effectively.

## 2 Results

### 2.1 Research subjects

**Figure 1:**
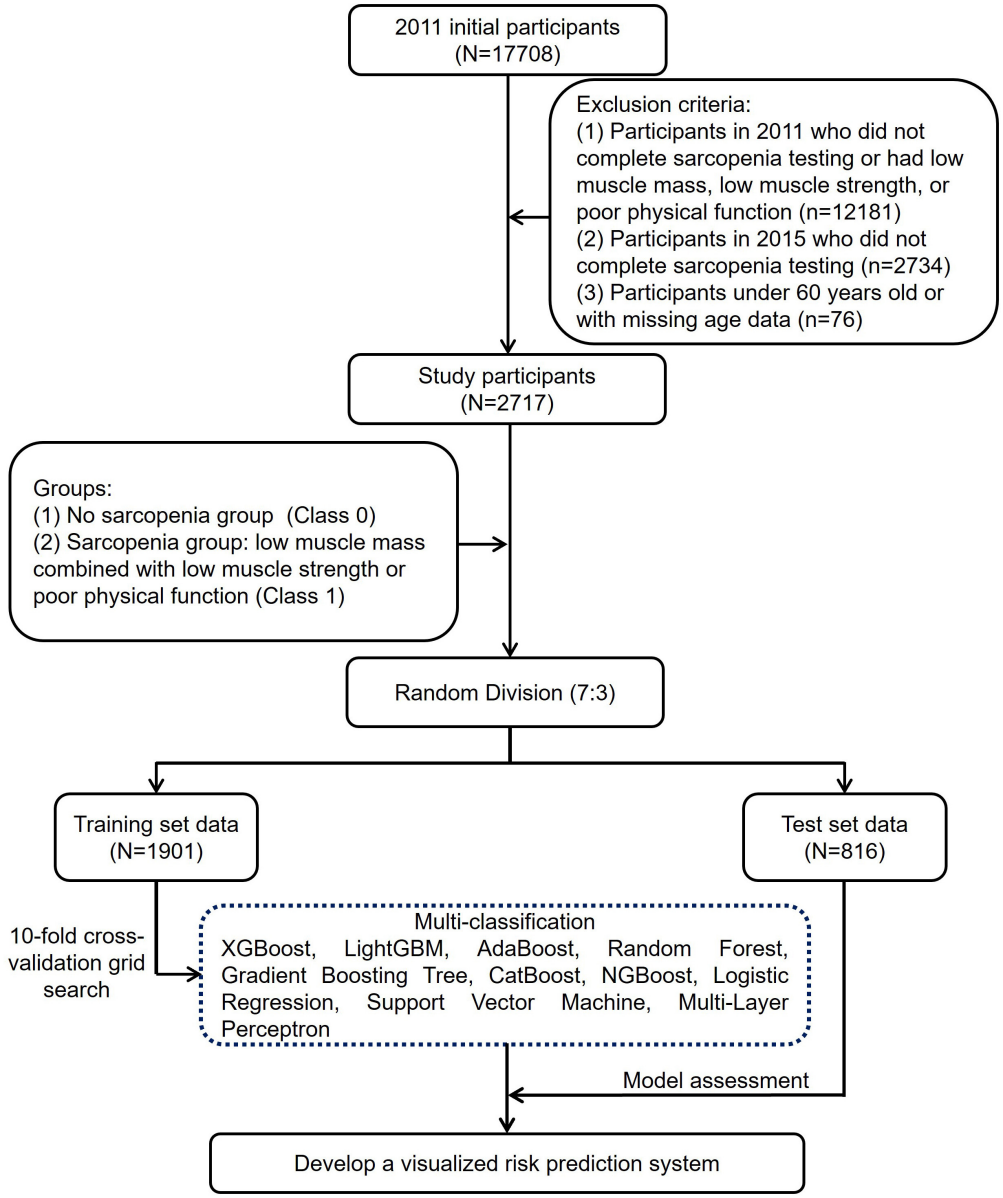
A flowchart describing the general framework of the study.

This study initially included 17,708 participants. After excluding 14,991 participants based on exclusion criteria, 2,717 participants remained (Figure 1). Among them, 1,397 were male (51.42%), and 1,320 were female (48.58%), with an average age of 66.25 ± 5.37 years (Supplementary Table S1). After a 4-year follow-up, 222 participants were diagnosed with sarcopenia in 2015, yielding a prevalence of 8.17%.

### 2.2 Classification performance

**Figure 2:**
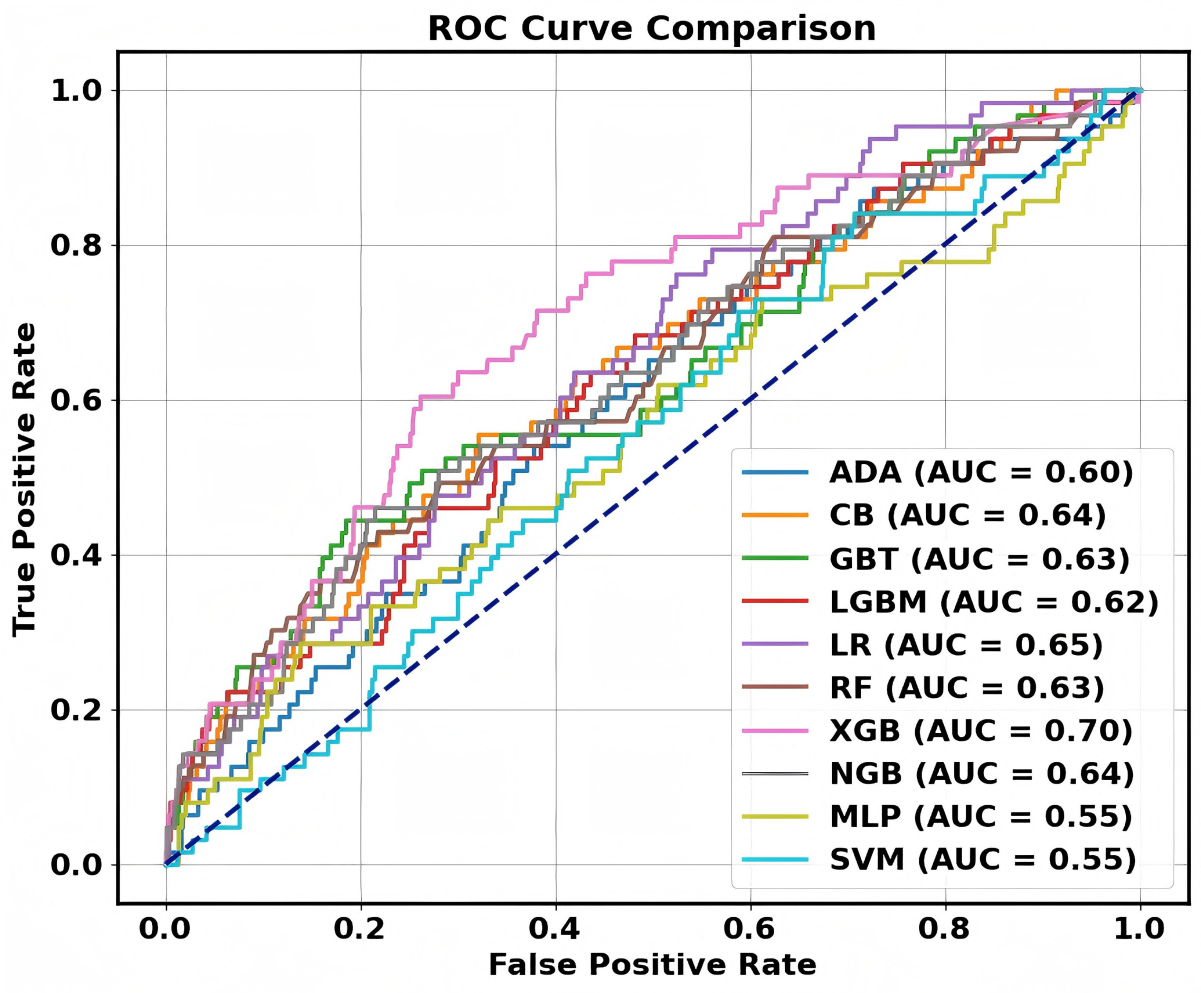
ROC curves for different machine learning models.

In this study, we built older adult sarcopenia risk assessment models using 10 algorithms, including XGBoost (XGB), LightGBM (LGBM), AdaBoost (ADA), Random Forest (RF), Gradient Boosting Trees (GBT), CatBoost (CB), NGBoost (NGB), Logistic Regression (LR), Multi-Layer Perceptron (MLP), and Support Vector Machine (SVM). We used accuracy, precision, F1-score, and the area under the receiver operating characteristic curve (ROC-AUC) as evaluation metrics. As shown in Figure 2, the models constructed using XGB, RF, and GBT algorithms showed higher ROC-AUC, possibly because these algorithms enhance model generalization through ensemble learning methods, while also demonstrating strong robustness and interpretability (14). Additionally, the XGB model outperformed other models in terms of F1-score (Supplementary Table S2), and with a ROC-AUC of 0.7, demonstrated significant performance advantages (Supplementary Table S3). This success is due to its highly optimized gradient boosting framework and flexible regularization mechanisms, which effectively mitigate overfitting and enhance the model's ability to capture complex data patterns (15). Based on the above results, the XGB model demonstrated high classification stability and reliability. Therefore, we ultimately selected the model built using the XGB algorithm as the older adult sarcopenia risk prediction model (Supplementary Code S1).

We analyzed the sensitivity of the ROC curve for the XGB model across different gender subgroups using the Hanley-McNeil test (Supplementary Figure S1 and Supplementary Code S2). The results showed (Supplementary Table S4) that the differences in ROC-AUC between the Overall Data, Male Subgroup, and Female Subgroup were not statistically significant. This indicates that the XGB model performs consistently across different gender subgroups. We further evaluated the classification performance of the XGB model using the confusion matrix (Supplementary Figure S2). Among the 753 participants who did not develop sarcopenia after 4 years, 73.84% were correctly classified; among the 63 participants who developed sarcopenia, 60.32% were correctly classified. This demonstrates that the XGB model has some sensitivity in predicting whether individuals will develop sarcopenia in the future.

### 2.3 Feature importance

SHAP plots are intuitive tools for interpreting machine learning model outputs, measuring each feature's contribution to predictions (16, 17). SHAP values indicate the direction and magnitude of a feature's influence on predictions: positive values indicate a positive impact, while negative values indicate a negative effect. In the SHAP plot, features are ranked by importance on the vertical axis, and their specific effects on model output are shown on the horizontal axis. Each point represents a sample, with color indicating feature values (red for high values and blue for low values), providing a clear visualization of the relationship between features and prediction outcomes.

**Figure 3:**
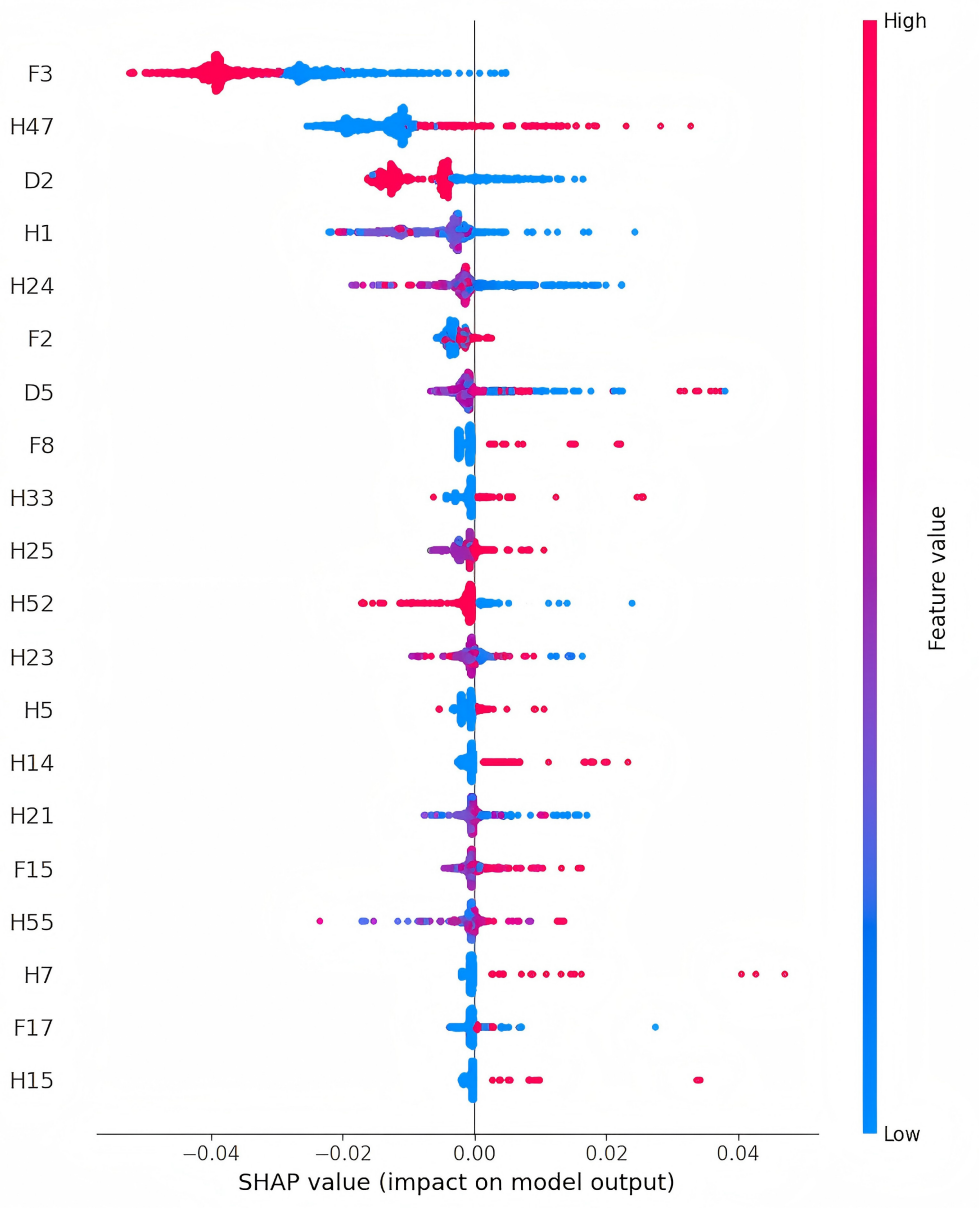
Feature importance charts for sarcopenia in older adults (The detailed description of the features can be found in Supplementary Table S1).

In the overall feature importance plot for sarcopenia (Figure 3), the household predictor “Does your residence have running water?” (F3) had the most significant impact, indicating a negative correlation between access to running water and future sarcopenia risk. Demographic predictors such as “Marital Status” (D2) and “Number of living children” (D5), and health predictors like “Have you lost all of your teeth?” (H47) and “The maximum value of the breathing test” (H24), also showed high importance. Participants with the following characteristics were at higher risk of developing sarcopenia: tooth loss (H47), poor self-rated health (H1), low breathing test values (H24), smoking (F2), lower systolic blood pressure (H21), and asthma (H14). These findings highlight the predictive value of living conditions, health status, and behavioral habits, providing a reference for targeted interventions and preventive strategies.

### 2.4 Risk prediction system

**Figure 4:**
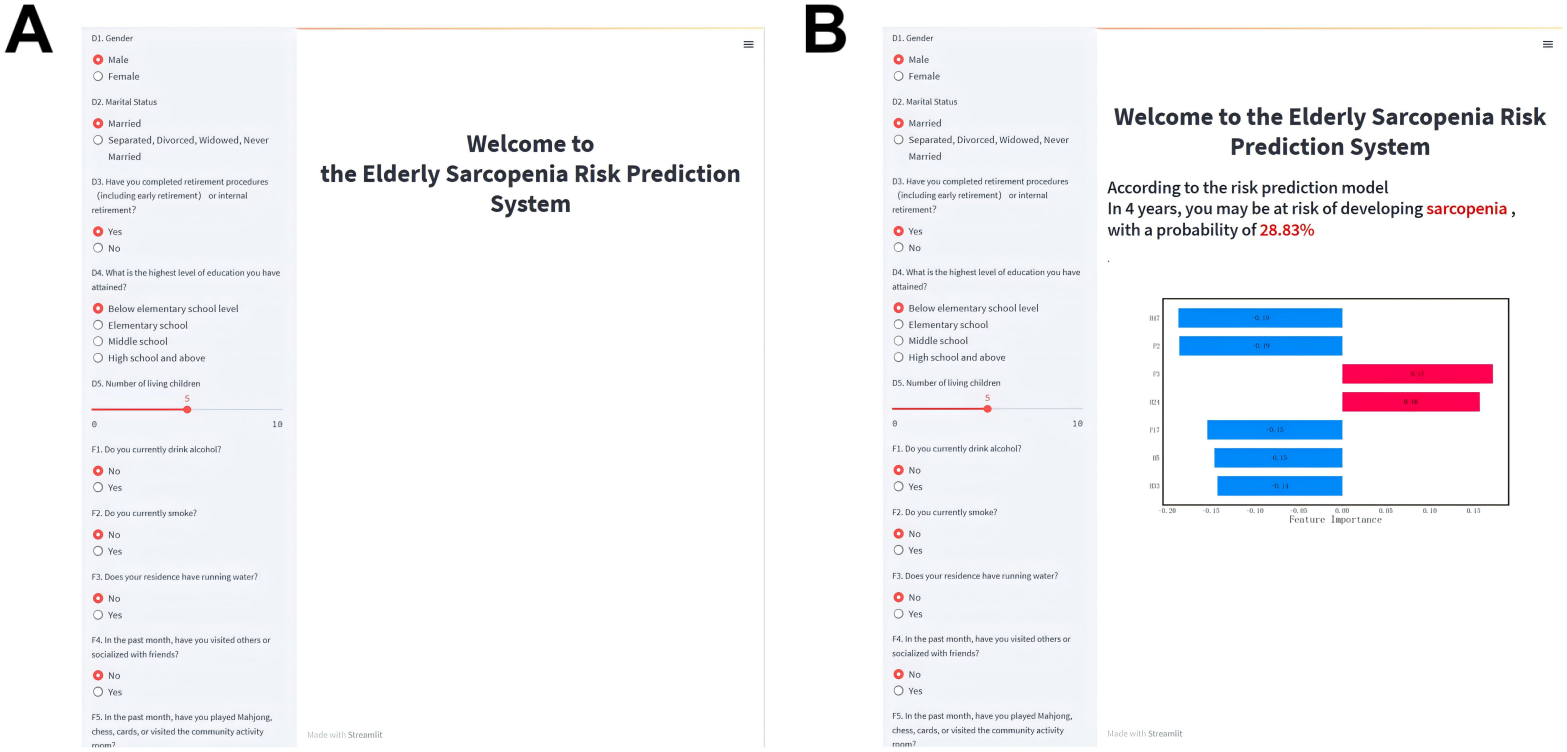
Visualized risk prediction system for older adults. **(A)** System homepage; **(B)** Information output page.

The visualized prediction system consists of an information input area on the left and a results display area on the right (Figure 4), shows that the input information is consistent with the features used in the training model (Supplementary Table S1). For continuous variables, input can be adjusted using sliders, while categorical variables (e.g., gender) can be selected by clicking. The right side of the interface is divided into two parts: the upper section displays the predicted sarcopenia status after 4 years, while the lower section provides a personalized analysis report to guide precise intervention strategies.

An example application of the prediction system is shown in Figure 4B. After entering relevant information on the left, the system predicts a 28.83% probability of developing sarcopenia within 4 years. Below, the SHAP plot visualizes how each feature influences the prediction. The length of the bars reflects the strength of the effect: red bars indicate positive influences, and blue bars indicate negative influences. Features positively associated with the prediction include F3 and H24, while H47, F2, F17, H5, and H33 had negative impacts. Based on this analysis, preventive measures such as installing running water facilities and improving maximum breathing values could reduce the user's future sarcopenia risk.

## 3 Discussion

This study successfully developed a sarcopenia risk prediction system for the older adult based on the CHARLS database using multiple machine learning algorithms. The system not only identifies high-risk individuals but also enhances usability and user experience through a visualized web interface, providing intuitive decision support for medical professionals.

Unlike previous studies that primarily relied on cross-sectional data (11–13), this study employed longitudinal data to conduct a cohort analysis of sarcopenia risk in the older adult. Cross-sectional studies often capture sarcopenia-related features at a single time point, limiting their ability to reflect dynamic changes and time-dependent risk factors during the disease progression. This limitation can lead to an incomplete understanding of the critical stages in sarcopenia development, reducing the accuracy and clinical applicability of predictive models. In contrast, longitudinal data enable the observation of individual characteristic trends over time, capturing dynamic processes and time-dependent risk factors, thus improving the predictive accuracy and clinical relevance of the model.

After comparing the performance of the models, the XGB model performed the best. In building the risk prediction model using the XGB algorithm, we employed the KNN algorithm for missing value imputation and used the SMOTE algorithm to address class imbalance. Although these methods may introduce some bias, the KNN algorithm, through its imputation strategy based on data similarity, better preserves the original structure and distribution of the data. Moreover, the SMOTE algorithm effectively alleviates class imbalance by generating synthetic samples, thereby improving the model's ability to recognize the minority class (18). To validate the effectiveness of these methods, we compared the performance of models under different preprocessing strategies. The experimental results showed that the model built using KNN imputation and SMOTE for data balancing had a significantly higher ROC-AUC than models using mean imputation, mode imputation, or no data balancing (Supplementary Figure S3). This result supports the rationale for choosing KNN and SMOTE during the preprocessing stage.

We selected the optimal F1-score threshold for prediction probability (Supplementary Code S3). This threshold selection method maximizes the F1-score, balancing the model's precision and recall. Clinically, the optimal F1-score threshold helps improve the diagnostic accuracy of sarcopenia while minimizing false positives and false negatives. According to the confusion matrix analysis of the XGB model (Supplementary Figure S2), among the 753 participants who did not develop sarcopenia after 4 years, the model correctly classified 73.84% of individuals, with a low false-positive rate, indicating that the model effectively avoids misclassifying healthy individuals as diseased, thus reducing unnecessary medical tests and treatment costs. However, among the 63 participants who developed sarcopenia after 4 years, the model correctly classified only 60.32%, with a relatively high false-negative rate. This may result in some patients who require treatment not being identified in time, missing the best opportunity for early intervention, potentially leading to worsening conditions and increasing the complexity and cost of subsequent treatments.

To enhance model interpretability, SHAP technology was used for visual explanations. Traditional machine learning models are often considered “black boxes” (19–21), with their decision-making processes and feature contributions remaining opaque. SHAP assigns contribution values to features, clarifying their roles in predictions. Analysis of SHAP values in this study revealed that features such as living conditions, health status, and behavioral habits significantly influenced the model's predictions. Among these, “Does your residence have running water?” (F3) had the most substantial impact, showing a negative correlation with sarcopenia risk. While no direct association between access to running water and sarcopenia risk has been established in existing studies, it is hypothesized that water, as an essential nutrient, plays a crucial role in overall health (22–24). This finding underscores the importance of improving infrastructure for promoting healthy aging and offers insights for public health policy. Additionally, “Have you lost all of your teeth?” (H47) was the second most influential feature in the model, suggesting that tooth loss is a significant indicator of sarcopenia risk. This aligns with studies such as Kusama et al. (25), which linked tooth loss to reduced protein intake, and Azzolino et al. (26), which found that poor oral health can influence food choices and nutritional intake, leading to frailty and sarcopenia. Other important predictive factors include lower breathing test values (H24) and smoking (F2). Research by Rahimi et al. (27) demonstrated that improved respiratory function significantly enhances muscle strength in older adult women, while Rom et al. (28) reported that smoking accelerates skeletal muscle loss through inflammation and oxidative stress. Consistent with the SHAP analysis results from the overall data, in different gender subgroups (Supplementary Figures S4 and S5), tap water (F3) and breathing test values (H24) have significant effects on the likelihood of sarcopenia in both men and women. However, the impact of alcohol consumption (F1) is greater for men, and marital status (D2) has a stronger influence on women regarding the risk of sarcopenia. These findings not only validate the model's predictions but also reveal potential mechanisms influencing sarcopenia. The developed system integrates the prediction model into a web platform, enabling users to input individual characteristics and generate personalized risk assessment reports based on SHAP analysis. This enhances accessibility and provides an intuitive understanding of the results.

However, the study has several limitations. First, the data were derived exclusively from Chinese older adult populations, potentially limiting its generalizability across different cultural or ethnic groups. Second, the model did not include certain potentially critical variables, such as genetic information, long-term exercise habits, and detailed dietary patterns, which may affect prediction accuracy. Additionally, although this study is based on longitudinal data from CHARLS, the temporal changes of predictive factors (such as the dynamic changes in health behaviors) were not adequately considered during model development, which may affect the long-term predictive ability of the model. Lastly, while SHAP technology elucidates the contributions of various features, the specific mechanisms linking certain features to sarcopenia remain underexplored. Future research should expand data sources, incorporate key variables such as genetic information, and optimize model performance. Experimental studies are also needed to further investigate the mechanisms between features and sarcopenia, providing scientific evidence for clinical interventions.

## 4 Conclusions

This study successfully developed a sarcopenia risk prediction model for the older adult based on the CHARLS database, enhancing its interpretability through SHAP technology and building a visualized web platform to make prediction results more accessible and applicable. The findings highlight the significant role of factors such as living conditions, dental health, and respiratory function in predicting sarcopenia risk, providing a foundation for policy development and clinical decision-making. However, the study has limitations, including the restricted data scope. Future efforts should aim to expand the sample range and integrate key variables to further optimize the model, offering more comprehensive support for precise sarcopenia prediction and promoting healthy aging.

## 5 Methods

### 5.1 Study population

This study utilized data from the China Health and Retirement Longitudinal Study (CHARLS), a multidisciplinary nationwide survey led by the National School of Development at Peking University. CHARLS covers 28 provinces, 150 counties, and 450 communities (villages) in China, involving ~10,000 households (29, 30). High-quality longitudinal data were collected through in-home visits from individuals aged 45 years and older. The study was approved by the Peking University Institutional Review Board (IRB00001052-11015), and all participants provided informed consent prior to participation. Ethical standards were strictly adhered to, ensuring transparency and integrity throughout the research process. As shown in Figure 1, the study sample was derived from the first (2011) and third (2015) waves of CHARLS. A total of 2,717 eligible participants were included in the final analysis after applying the following exclusion criteria: (1) Participants in 2011 who did not complete sarcopenia testing or had low muscle mass, low muscle strength, or poor physical function; (2) Participants in 2015 who did not complete sarcopenia testing; (3) Participants under 60 years old or with missing age data.

### 5.2 Research variables

Sarcopenia was assessed based on the Asian Working Group for Sarcopenia (AWGS 2019) criteria, encompassing three components: muscle strength, appendicular skeletal muscle mass (ASM), and physical function (31). Muscle strength was evaluated by measuring handgrip strength using a Yuejian™ WL-1000 hand dynamometer (Nantong Yuejian Measuring Instruments Co., Ltd., Nantong, China). Participants were instructed to squeeze the dynamometer with maximum effort, and two measurements were taken for both the dominant and non-dominant hands. The maximum grip strength was recorded, and the average was used. According to AWGS 2019, the thresholds for low grip strength are < 28 kg for men and < 18 kg for women. ASM was estimated using a validated body composition formula (Supplementary Equation S1), which has been shown to correlate strongly with dual-energy X-ray absorptiometry (DXA) results (32, 33). ASM adjusted for height squared (ASM/Ht^2^) was calculated by dividing ASM by the square of height in meters. Low muscle mass was defined as the lowest 20th percentile of height-adjusted muscle mass in the study population: ASM/Ht^2^ < 4.90 kg/m^2^ for women and < 6.79 kg/m^2^ for men. Physical function was assessed by gait speed, measured as the time taken to walk 2.5 m back and forth at a normal pace. Low physical function was defined as gait speed < 1.0 m/s based on AWGS 2019 recommendations. Participants were classified into two groups: non-sarcopenia and sarcopenia, with the latter defined as low muscle mass combined with either low muscle strength or low physical function. This study included 78 feature variables (Supplementary Table S1), with missing data rates below 10% for all variables. Given the strong correlation between age and muscle mass, the remaining 77 features were used as predictors, encompassing three categories: demographics, family lifestyle, and health status. Demographic variables included five features (excluding age), such as sex and marital status; family lifestyle included 17 variables, such as current smoking and alcohol consumption; and health status included 55 variables, such as self-rated health and bodily pain.

### 5.3 Prediction system development

A visualized risk assessment system for sarcopenia in older adults was developed using Python 3.11. The dataset was randomly split into training and testing sets at a 7:3 ratio. Missing data were imputed using the K-Nearest Neighbors (KNN) algorithm, and the Synthetic Minority Oversampling Technique (SMOTE) was employed on the training set to address class imbalance (18). We selected the optimal hyperparameters through 10-fold cross-validation with grid search and constructed XGB, LGBM, ADA, RF, GBT, CB, NGB, LR, MLP, and SVM models. Optimal hyperparameters were determined through grid search with 10-fold cross-validation. Eight models—XGB, LGBM, ADA, RF, GBT, CB, NGB, and LR—were constructed, with performance metrics including accuracy, precision, F1-score, and area under the ROC curve. The model with the best performance was selected as the sarcopenia risk assessment model. Feature importance indices were calculated using SHapley Additive exPlanations (SHAP), and an online risk assessment system was developed. Additionally, to validate the effect of data processing, we also applied mean imputation, mode imputation, and no data balancing strategies, using the best model construction algorithms.

## Data Availability

Publicly available datasets were analyzed in this study. This data can be found here: https://charls.charlsdata.com/pages/data/111/zh-cn.html.
