# Supplementary material for: Development of a visualized risk prediction system for sarcopenia in older adults using machine learning: a cohort study based on CHARLS
Source: Front Public Health. 2025 Mar 12;13:1544894. doi: 10.3389/fpubh.2025.1544894 (PMC11936879; doi:10.3389/fpubh.2025.1544894)
Supplement: Supplementary file 1 [file Data_Sheet_1.docx]

Supplementary Material

Development of a Visualized Risk Prediction System for Sarcopenia in Older Adults Using Machine Learning: A Cohort Study Based on CHARLS

**Supplementary Tables**

Table S1. Baseline Characteristics of the Study Population.

| **Variables** | **Distribution** |  |
| --- | --- | --- |
| **Sarcopenia** | |  |
| **Class 0.** No sarcopenia | 2495 (91.83%) |  |
| **Class 1.** Sarcopenia | 222 (8.17%) |  |
| **Demographic** | |  |
| Age | 66.25 (5.37)* |  |
| **D1.** Gender |  |  |
| Male (1) | 1397 (51.42%) |  |
| Female (0) | 1320 (48.58%) |  |
| **D2.** Marital Status |  |  |
| Married (1) | 2260 (83.18%) |  |
| Separated, Divorced, Widowed, Never Married (0) | 457 (16.82%) |  |
| **D3.** Have you completed retirement procedures （including early retirement） or internal retirement？ |  |  |
| Yes (1) | 420 (15.46%) |  |
| No (2) | 2241 (82.48%) |  |
| **D4.** What is the highest level of education you have attained? |  |  |
| Below elementary school level (1) | 1450 (53.37%) |  |
| Elementary school (2) | 759 (27.94%) |  |
| Middle school (3) | 354 (13.03%) |  |
| High school and above (4) | 153 (5.63%) |  |
| **D5.** Number of living children | 3.23 (1.49)* |  |
| **Family Life** | |  |
| **F1.** Do you currently drink alcohol? |  |  |
| No (0) | 1841 (67.76%) |  |
| Yes (1) | 875 (32.20%) |  |
| **F2.** Do you currently smoke? |  |  |
| No (0) | 1831 (67.39%) |  |
| Yes (1) | 874 (32.17%) |  |
| **F3.** Does your residence have running water? |  |  |
| No (0) | 1091 (40.15%) |  |
| Yes (1) | 1620 (59.62%) |  |
| **F4.** In the past month, have you visited others or socialized with friends? |  |  |
| No (0) | 1759 (64.74%) |  |
| Yes (1) | 938 (34.52%) |  |
| **F5.** In the past month, have you played Mahjong, chess, cards, or visited the community activity room? |  |  |
| No (0) | 2197 (80.86%) |  |
| Yes (1) | 500 (18.40%) |  |
| **F6.** In the past month, have you provided help to relatives, friends, or neighbors who do not live with you? |  |  |
| No (0) | 2537 (93.38%) |  |
| Yes (1) | 160 (5.89%) | |
| **F7.** In the past month, have you participated in activities like dancing, fitness exercises, or practicing qigong? |  |  |
| No (0) | 2543 (93.60%) |  |
| Yes (1) | 154 (5.67%) |  |
| **F8.** In the past month, have you participated in activities organized by any clubs or associations? |  |  |
| No (0) | 2652 (97.61%) |  |
| Yes (1) | 45 (1.66%) |  |
| **F9.** In the past month, have you participated in any volunteer or charitable activities? |  |  |
| No (0) | 2684 (98.79%) |  |
| Yes (1) | 13 (0.48%) |  |
| **F10.** In the past month, have you cared for any sick or disabled individuals who do not live with you? |  |  |
| No (0) | 2681 (98.68%) |  |
| Yes (1) | 16 (0.59%) |  |
| **F11.** In the past month, have you attended school or participated in any training courses? |  |  |
| No (0) | 2694 (99.15%) |  |
| Yes (1) | 3 (0.11%) |  |
| **F12.** In the past month, have you traded stocks? |  |  |
| No (0) | 2693 (99.12%) |  |
| Yes (1) | 4 (0.15%) |  |
| **F13.** In the past month, have you used the internet? |  |  |
| No (0) | 2673 (98.38%) |  |
| Yes (1) | 24 (0.88%) |  |
| **F14.** Are you hopeful about the future? |  |  |
| Rarely or none of the time (<1 day) (1) | 660 (24.29%) |  |
| Some or a little of the time (1-2 days) (2) | 442 (16.27%) |  |
| Occasionally or a moderate amount of the time (3-4 days) (3) | 514 (18.92%) |  |
| Most or all of the time (5-7 days) (4) | 1006 (37.03%) |  |
| **F15.** During the past month, how many hours of actual sleep did you get at night (average hours for one night)? | 6.24 (1.93)* |  |
| **F16.** During the past month, how long did you take a nap after lunch? | 34.75 (43.77)* |  |
| **F17.** How many meals do you normally eat every day? | 3.11 (0.4)* |  |
| **Health Status** | |  |
| **H1.** Would you say your health is excellent？ |  |  |
| Excellent (1) | 92 (3.39%) |  |
| Very good (2) | 635 (23.37%) |  |
| Good (3) | 1402 (51.60%) |  |
| Fair (4) | 425 (15.64%) |  |
| Poor (5) | 162 (5.96%) |  |
| **H2.** Has a doctor ever told you that you have high blood pressure? |  |  |
| No (0) | 1864 (68.61%) |  |
| Yes (1) | 845 (31.10%) |  |
| **H3.** Has a doctor ever told you that you have diabetes? |  |  |
| No (0) | 2523 (92.86%) |  |
| Yes (1) | 177 (6.51%) |  |
| **H4.** Has a doctor ever told you that you have cancer? |  |  |
| No (0) | 2683 (98.75%) |  |
| Yes (1) | 19 (0.70%) |  |
| **H5.** Has a doctor ever told you that you have lung disease? |  |  |
| No (0) | 2370 (87.23%) |  |
| Yes (1) | 339 (12.48%) |  |
| **H6.** Has a doctor ever told you that you have heart disease? |  |  |
| No (0) | 2323 (85.50%) |  |
| Yes (1) | 377 (13.88%) |  |
| **H7.** Has a doctor ever told you that you have had a stroke? |  |  |
| No (0) | 2652 (97.61%) |  |
| Yes (1) | 60 (2.21%) |  |
| **H8.** Has a doctor ever told you that you have a mental illness? |  |  |
| No (0) | 2664 (98.05%) |  |
| Yes (1) | 38 (1.40%) |  |
| **H9.** Has a doctor ever told you that you have arthritis? |  |  |
| No (0) | 1737 (63.93%) |  |
| Yes (1) | 976 (35.92%) |  |
| **H10.** Has a doctor ever told you that you have abnormal blood lipid levels? |  |  |
| No (0) | 2402 (88.41%) |  |
| Yes (1) | 273 (10.05%) |  |
| **H11.** Has a doctor ever told you that you have liver disease? |  |  |
| No (0) | 2613 (96.17%) |  |
| Yes (1) | 87 (3.20%) |  |
| **H12.** Has a doctor ever told you that you have kidney disease? |  |  |
| No (0) | 2542 (93.56%) |  |
| Yes (1) | 159 (5.85%) |  |
| **H13.** Has a doctor ever told you that you have a stomach disease? |  |  |
| No (0) | 2125 (78.21%) |  |
| Yes (1) | 584 (21.49%) |  |
| **H14.** Has a doctor ever told you that you have asthma? |  |  |
| No (0) | 2545 (93.67%) |  |
| Yes (1) | 163 (6.00%) |  |
| **H15.** Has a doctor ever told you that you have a memory disorder? |  |  |
| No (0) | 2665 (98.09%) |  |
| Yes (1) | 43 (1.58%) |  |
| **H16.** How many times have you received inpatient care during the past year? | 0.12 (0.53)* |  |
| **H17.** The number of hospitalization days in the past year. | 0.95 (3.75)* |  |
| **H18.** The number of outpatient visits in the past month. | 0.44 (1.41)* |  |
| **H19.** Total hospitalization expenses. | 540.71 (3693.11)* |  |
| **H20.** Total outpatient expenses. | 80.39 (492.1)* |  |
| **H21.** Mean systolic blood pressure | 133.27 (21.31)* |  |
| **H22.** Mean diastolic blood pressure | 74.65 (11.34)* |  |
| **H23.** Mean pulse rate | 71.6 (10.37)* |  |
| **H24.** The maximum value of the breathing test | 275.32 (118.2)* |  |
| **H25.** Please think about your life-as-a-whole. How satisfied are you with it? |  |  |
| Completely satisfied (1) | 50 (1.84%) |  |
| Very satisfied (2) | 277 (10.20%) |  |
| Somewhat satisfied (3) | 1504 (55.36%) |  |
| Not very satisfied (4) | 607 (22.34%) |  |
| Not at all satisfied (5) | 66 (2.43%) |  |
| **H26.** Does your head feel painful? |  |  |
| No (0) | 2378 (87.52%) |  |
| Yes (1) | 334 (12.29%) |  |
| **H27.** Does your shoulder feel painful? |  |  |
| No (0) | 2385 (87.78%) |  |
| Yes (1) | 327 (12.04%) |  |
| **H28.** Does your arm feel painful? |  |  |
| No (0) | 2453 (90.28%) |  |
| Yes (1) | 259 (9.53%) |  |
| **H29.** Does your wrist feel painful |  |  |
| No (0) | 2556 (94.07%) |  |
| Yes (1) | 156 (5.74%) |  |
| **H30.** Do your fingers feel painful? |  |  |
| No (0) | 2555 (94.04%) |  |
| Yes (1) | 157 (5.78%) |  |
| **H31.** Does your chest feel painful? |  |  |
| No (0) | 2546 (93.71%) |  |
| Yes (1) | 166 (6.11%) |  |
| **H32.** Does your stomach feel painful? |  |  |
| No (0) | 2487 (91.53%) |  |
| Yes (1) | 225 (8.28%) |  |
| **H33.** Does your back feel painful? |  |  |
| No (0) | 2459 (90.50%) |  |
| Yes (1) | 253 (9.31%) |  |
| **H34.** Does your lower back feel painful? |  |  |
| No (0) | 2174 (80.01%) |  |
| Yes (1) | 538 (19.80%) |  |
| **H35.** Does your hip feel painful? |  |  |
| No (0) | 2608 (95.99%) |  |
| Yes (1) | 104 (3.83%) |  |
| **H36.** Does your leg feel painful? |  |  |
| No (0) | 2289 (84.25%) |  |
| Yes (1) | 423 (15.57%) |  |
| **H37.** Does your knee feel painful? |  |  |
| No (0) | 2316 (85.24%) |  |
| Yes (1) | 396 (14.57%) |  |
| **H38.** Does your ankle feel painful? |  |  |
| No (0) | 2555 (94.04%) |  |
| Yes (1) | 157 (5.78%) |  |
| **H39.** Do your toes feel painful? |  |  |
| No (0) | 2621 (96.47%) |  |
| Yes (1) | 91 (3.35%) |  |
| **H40.** Does your neck feel painful? |  |  |
| No (0) | 2535 (93.30%) |  |
| Yes (1) | 177 (6.51%) |  |
| **H41.** Have you fallen down in the last two years? |  |  |
| No (0) | 2228 (82.00%) |  |
| Yes (1) | 469 (17.26%) |  |
| **H42.** Do you usually wear glasses or corrective lenses? |  |  |
| No (0) | 2313 (85.13%) |  |
| Yes (1) | 397 (14.61%) |  |
| Blindness (2) | 6 (0.22%) |  |
| **H43.** How good is your eyesight for seeing things at a distance？ |  |  |
| Excellent (1) | 711 (26.17%) |  |
| Very good (2) | 1112 (40.93%) |  |
| Good (3) | 593 (21.83%) |  |
| Fair (4) | 250 (9.20%) |  |
| Poor (5) | 30 (1.10%) |  |
| **H44.** How good is your eyesight for seeing things up close？ |  |  |
| Excellent (1) | 64 (2.36%) |  |
| Very good (2) | 1234 (45.42%) |  |
| Good (3) | 621 (22.86%) |  |
| Fair (4) | 197 (7.25%) |  |
| Poor (5) | 21 (0.77%) |  |
| **H45.** Do you ever wear a hearing aid? |  |  |
| No (0) | 2701 (99.41%) |  |
| Yes (1) | 15 (0.55%) |  |
| **H46.** How is your hearing? |  |  |
| Excellent (1) | 477 (17.56%) |  |
| Very good (2) | 1194 (43.95%) |  |
| Good (3) | 714 (26.28%) |  |
| Fair (4) | 301 (11.08%) |  |
| Poor (5) | 29 (1.07%) |  |
| **H47.** Have you lost all of your teeth? |  |  |
| No (0) | 2289 (84.25%) |  |
| Yes (1) | 423 (15.57%) |  |
| **H48.** Have you ever fractured your hip? |  |  |
| No (0) | 2657 (97.79%) |  |
| Yes (1) | 39 (1.44%) |  |
| **H49.** Do you have a physical disability? |  |  |
| No (0) | 2639 (97.13%) |  |
| Yes (1) | 75 (2.76%) |  |
| **H50.** Do you have brain damage/intellectual disabilities? |  |  |
| No (0) | 2649 (97.50%) |  |
| Yes (1) | 65 (2.39%) |  |
| **H51.** Are you blind or partially sighted? |  |  |
| No (0) | 2498 (91.94%) |  |
| Yes (1) | 217 (7.99%) |  |
| **H52.** Are you deaf or hard of hearing? |  |  |
| No (0) | 2384 (87.74%) |  |
| Yes (1) | 332 (12.22%) |  |
| **H53.** Are you mute or severely stutter? |  |  |
| No (0) | 2709 (99.71%) |  |
| Yes (1) | 3 (0.11%) |  |
| **H54.** Are you often troubled with any body pains? |  |  |
| No (0) | 1798 (66.18%) |  |
| Yes (1) | 917 (33.75%) |  |
| **H55.** How would you evaluate your health during childhood, up to and including age 15? |  |  |
| Excellent (1) | 263 (9.68%) |  |
| Very good (2) | 992 (36.51%) |  |
| Good (3) | 730 (26.87%) |  |
| Fair (4) | 513 (18.88%) |  |
| Poor (5) | 202 (7.43%) |  |

Table S2. Accuracy, Precision, Recall and F1-Score of the Eight Machine Learning Models.

|  | XGB | LGBM | ADA | RF | GBT | CB | NGB | LR | MLP | SVM |
| --- | --- | --- | --- | --- | --- | --- | --- | --- | --- | --- |
| Accuracy | 0.7279 | 0.8934 | 0.6066 | 0.8456 | 0.875 | 0.8811 | 0.761 | 0.848 | 0.8211 | 0.4473 |
| Precision | 0.1617 | 0.26 | 0.1138 | 0.1881 | 0.2254 | 0.2258 | 0.1526 | 0.1789 | 0.1513 | 0.0855 |
| Recall | 0.6032 | 0.2063 | 0.6032 | 0.3016 | 0.254 | 0.2222 | 0.4603 | 0.2698 | 0.2857 | 0.6349 |
| F1-score | 0.255 | 0.23 | 0.1914 | 0.2317 | 0.2388 | 0.2171 | 0.224 | 0.2152 | 0.1978 | 0.1507 |

Table S3. Hanley-McNeil Test for ROC-AUC of Ten Machine Learning Models.

|  | XGB | LGBM | ADA | RF | GBT | CB | NGB | LR | MLP | SVM |
| --- | --- | --- | --- | --- | --- | --- | --- | --- | --- | --- |
| XGB |  |  |  |  |  |  |  |  |  |  |
| LGBM | 0.002 |  |  |  |  |  |  |  |  |  |
| ADA | 0.001> | 0.249 |  |  |  |  |  |  |  |  |
| RF | 0.005 | 0.82 | 0.168 |  |  |  |  |  |  |  |
| GBT | 0.007 | 0.731 | 0.135 | 0.907 |  |  |  |  |  |  |
| CB | 0.012 | 0.588 | 0.09 | 0.753 | 0.842 |  |  |  |  |  |
| NGB | 0.011 | 0.615 | 0.098 | 0.782 | 0.873 | 0.969 |  |  |  |  |
| LR | 0.053 | 0.263 | 0.023 | 0.372 | 0.437 | 0.563 | 0.537 |  |  |  |
| MLP | 0.001> | 0.004 | 0.079 | 0.002 | 0.001> | 0.001> | 0.001> | 0.001> |  |  |
| SVM | 0.001> | 0.001> | 0.04 | 0.001> | 0.001> | 0.001> | 0.001> | 0.001> | 0.766 |  |

Table S4. Hanley-McNeil Test for ROC-AUC of Overall Data and Different Gender Subgroups.

|  | *Z* | *p* |
| --- | --- | --- |
| Male Subgroup | 1.273 | 0.203 |
| Female Subgroup | 1.045 | 0.296 |

**Supplementary Figure**

Figure S1. ROC Curves for Overall Data and Different Gender Subgroups.


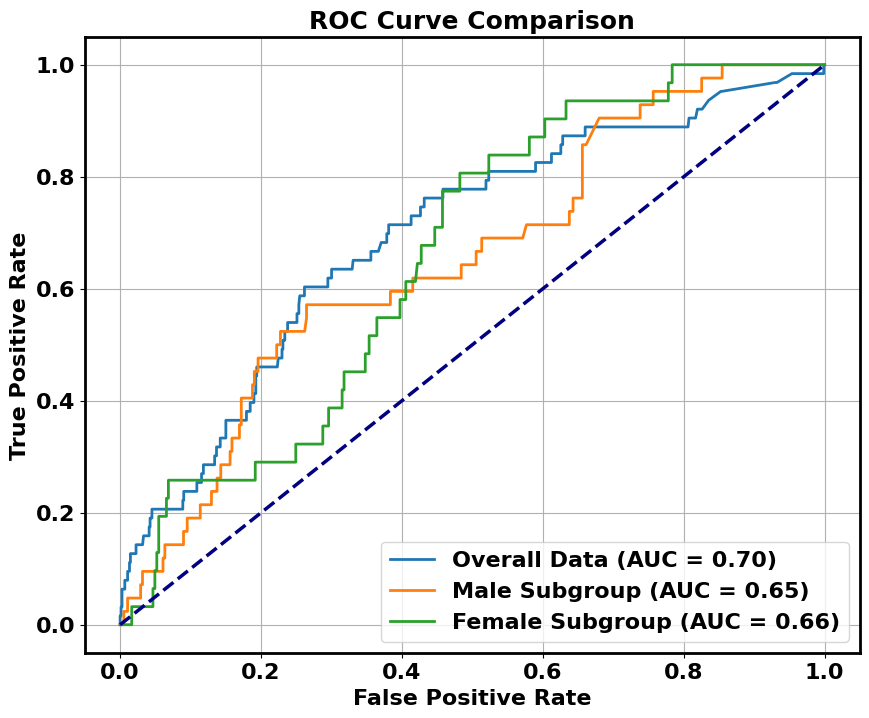


Figure S2. The mix matrix of the XGB model classification results based on the test set.


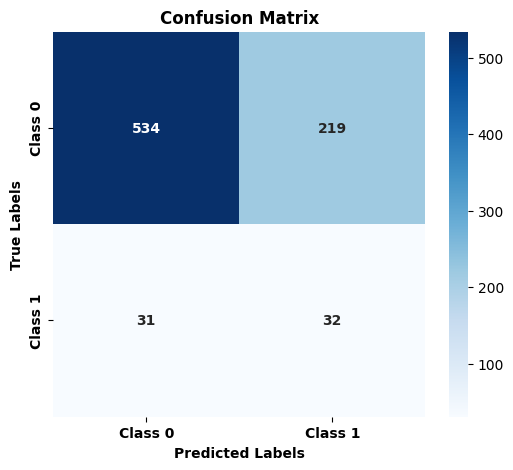


Figure S3. ROC Curves for Models under Different Preprocessing Strategies.


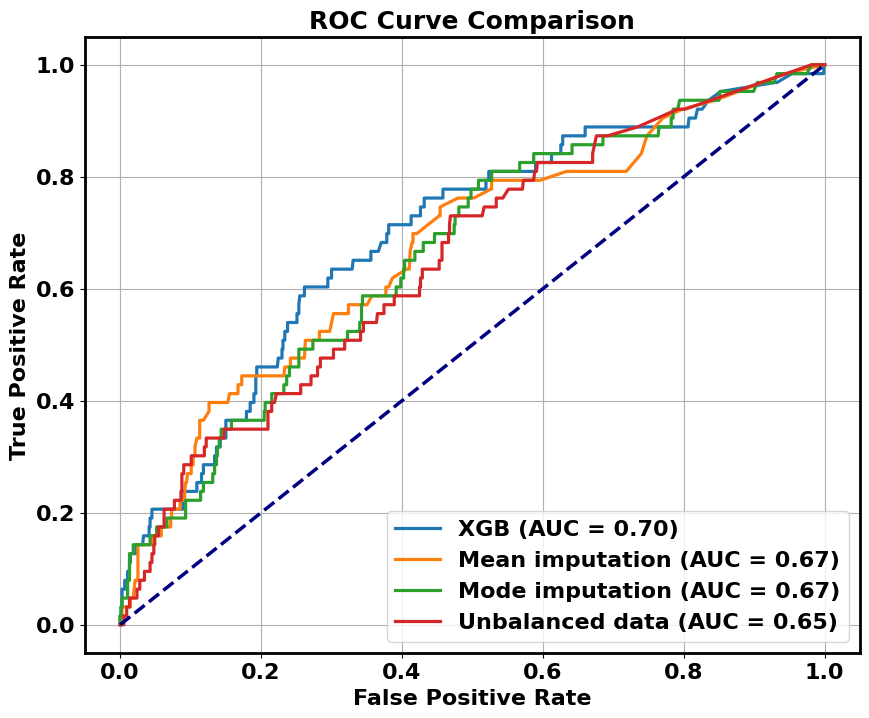


Figure S4. SHAP Plot for the Male Subgroup.


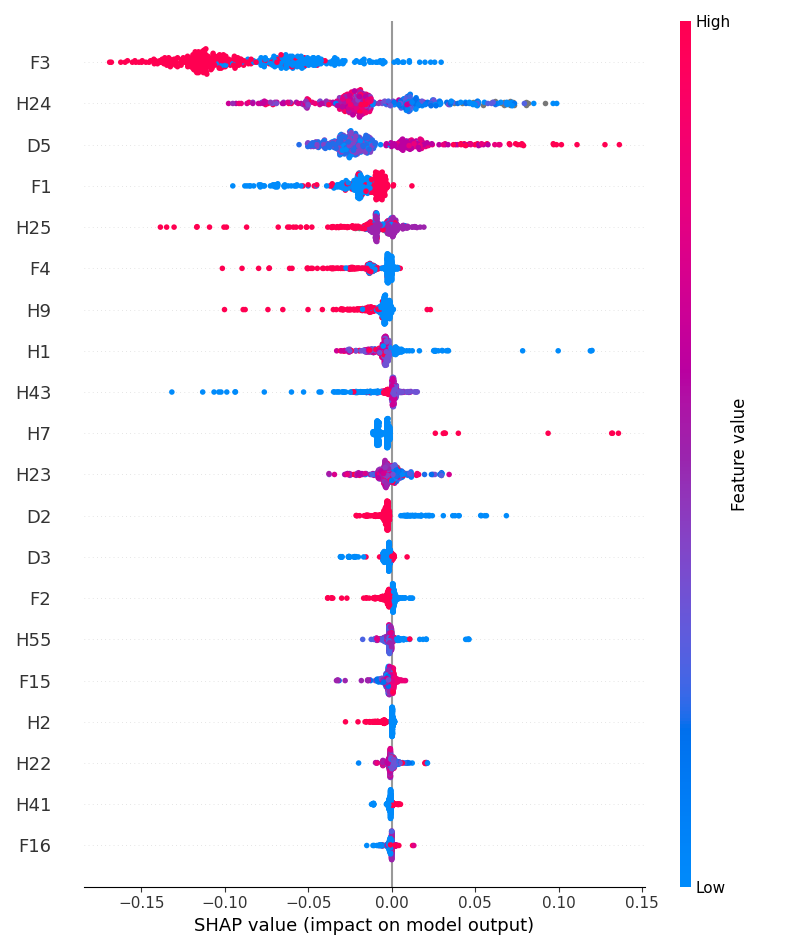


Figure S5. SHAP Plot for the Female Subgroup.


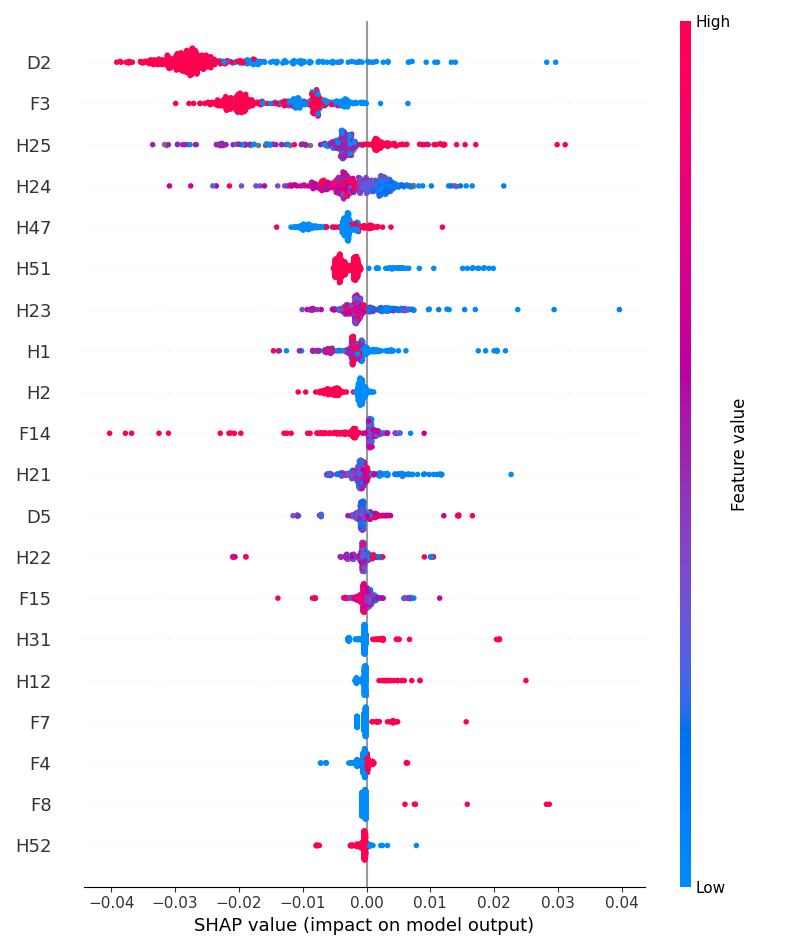


**Supplementary Equation**

Equation S1. Estimation formula for appendicular skeletal muscle (ASM) mass.

$$ASM=0.193*weight(kg)+0.107*height(cm)-4.157*sex(1 for male, 2 for female)-0.037*age-2.631$$

**Supplementary Code**

Code S1. Core Code for Sarcopenia Risk Prediction Model in Elderly.

| *param_grid = {*  *'n_estimators': [1000],*  *'max_depth': [70],*  *'learning_rate': [0.00088],*  *'gamma': [0.01],*  *'colsample_bytree': [0.945],*  *'min_child_weight': [2]*  *}*  *xgb_model = XGBClassifier()* |
| --- |

Code S2. Core Code for Hanley-McNeil Test.

| *def hanley_mcneil_test(auc_1, auc_2, n):*  *se_1 = np.sqrt((auc_1 * (1 - auc_1)) / n)*  *se_2 = np.sqrt((auc_2 * (1 - auc_2)) / n)*  *z_value = (auc_1 - auc_2) / np.sqrt(se_1 ** 2 + se_2 ** 2)*  *p_value = 2 * (1 - norm.cdf(np.abs(z_value)))*  *return z_value, p_value*  *auc_scores = calculate_auc(model_pred_probs, model_true_labels)*  *model_names = list(auc_scores.keys())*  *for i in range(len(model_names)):*  *for j in range(i + 1, len(model_names)):*  *model_1 = model_names[i]*  *model_2 = model_names[j]*  *auc_model_1 = auc_scores[model_1]*  *auc_model_2 = auc_scores[model_2]*  *z_value, p_value = hanley_mcneil_test(auc_model_1, auc_model_2, len(y_test))*  *print(f"Hanley-McNeil：{model_1} and {model_2}:")*  *print(f"Z: {z_value:.3f}, p: {p_value:.3f}")* |
| --- |

Code S3. Core Code for Determining Threshold Based on Optimal F1-Score.

| *best_f1 = 0*  *best_threshold = 0*  *for threshold in np.arange(0.0, 1.0, 0.0001):*  *y_pred = (y_pred_proba[:, 1] >= threshold).astype(int)*  *f1 = f1_score(y_test, y_pred)*  *if f1 > best_f1:*  *best_f1 = f1*  *best_threshold = threshold*  *y_pred = (y_pred_proba[:, 1] >= best_threshold).astype(int)* |
| --- |
